# Supplementary material for: A fault-intrusion-tolerant system and deadline-aware algorithm for scheduling scientific workflow in the cloud
Source: PeerJ Comput Sci. 2021 Nov 2;7:e747. doi: 10.7717/peerj-cs.747 (PMC8576554; doi:10.7717/peerj-cs.747)
Supplement: Supplemental Information 6 [file peerj-cs-07-747-s006.zip › Myproject1.1/docs/org/workflowsim/clustering/balancing/metrics/BalancingMetric.html]

BalancingMetric


JavaScript is disabled on your browser.


- Overview
- Package
- Class
- Use
- Tree
- Deprecated
- Index
- Help

- Prev Class
- Next Class

- Frames
- No Frames

- All Classes

- Summary:
- Nested |
- Field |
- Constr |
- Method

- Detail:
- Field |
- Constr |
- Method


org.workflowsim.clustering.balancing.metrics

## Interface BalancingMetric

- All Known Implementing Classes:
  :   DistanceVariance, HorizontalRuntimeVariance, ImpactFactorVariance, PipelineRuntimeVariance

  ---

    

  ```
  public interface BalancingMetric
  ```

  Every balancing metric should implement this interface

  Since:
  :   WorkflowSim Toolkit 1.0

- - ### Method Summary

    Methods

    | Modifier and Type | Method and Description |
    | --- | --- |
    | `double` | `getMetric(java.util.ArrayList<TaskSet> list)` Gets the metric value. |

- - ### Method Detail


    - #### getMetric

      ```
      double getMetric(java.util.ArrayList<TaskSet> list)
      ```

      Gets the metric value.


- Overview
- Package
- Class
- Use
- Tree
- Deprecated
- Index
- Help

- Prev Class
- Next Class

- Frames
- No Frames

- All Classes

- Summary:
- Nested |
- Field |
- Constr |
- Method

- Detail:
- Field |
- Constr |
- Method
